# D609 protects retinal pigmented epithelium as a potential therapy for age-related macular degeneration

**DOI:** 10.1038/s41392-020-0122-1

**Published:** 2020-03-04

**Authors:** Bowen Wang, Li Wang, Sijie Gu, Yankun Yu, Huaxing Huang, Kunlun Mo, He Xu, Fanzhu Zeng, Yichen Xiao, Lulu Peng, Chunqiao Liu, Nan Cao, Yizhi Liu, Jin Yuan, Hong Ouyang

**Affiliations:** 1 State Key Laboratory of Ophthalmology, Zhongshan Ophthalmic Center, Sun Yat-sen University, Guangzhou, 510623 China; 2 Program of Stem Cells and Regenerative Medicine, Fifth Affiliated Hospital, Zhongshan School of Medicine, Sun Yat-Sen University, Guangdong, 510080 China

**Keywords:** Drug screening, Diseases

## Abstract

Accumulated oxidative damage may lead to irreversible retinal pigmented epithelium (RPE) cell death, which is considered to be the primary cause of dry age-related macular degeneration (AMD), leading to blindness in the elderly. However, an effective therapy for this disease is lacking. Here, we described a robust high-content screening procedure with a library of 814 protective compounds and found that D609 strongly protected RPE cells from sodium iodate (SI)-induced oxidative cell death and prolonged their healthy survival. D609 effectively attenuated excessive reactive oxygen species (ROS) and prevented severe mitochondrial loss due to oxidative stress in the RPE cells. Surprisingly, the potent antioxidative effects of D609 were not achieved through its own reducibility but were primarily dependent on its ability to increase the expression of metallothionein. The injection of this small water-soluble molecule also showed an explicit protective effect of the RPE layer in an SI-induced AMD mouse model. These findings suggested that D609 could serve as a novel antioxidative protector of RPE cells both in vitro and in vivo and unveiled a novel antioxidative mechanism of D609, which may ultimately have clinical applications for the treatment of AMD.

## Introduction

The leading cause of irreversible vision disability among the elderly population is age-related macular degeneration (AMD), which is characterized by gradual central vision loss, straight-line blurring, and decreasing color recognition. Currently, 30–50 million people are affected by AMD worldwide, and the number of patients is expected to double in the following three decades due to increasing life expectancy.^1^ Up to 90% of all AMD patients suffer from the more prevalent dry (atrophic) form, as opposed to the wet (exudative) form of AMD.^2^ Aging is the top risk factor for AMD, as this irreversible process is accompanied by accumulated oxidative stress, mitochondrial dysfunction, and chronic inflammation.^3^ The complexity of aging makes the underlying pathogenesis of dry AMD difficult to clarify and makes this disease largely untreatable.

In advanced dry AMD—characterized by geographic atrophy—the most distinguished event is the degeneration or death of retinal pigmented epithelium (RPE) cells.^4,5^ RPE is a monolayer of pigmented cells located between the neural retina and the choroid that mainly function as a retinal blood barrier and scavenger of the outersegments of photoreceptors.^6^ In normal vision, active retinal metabolism consumes a high level of oxygen,^7^ which exposes RPE cells to concomitant reactive oxygen species (ROS).^8^ Accumulated or acute instant oxidative stress leads to RPE oxidative cell damage, including multicomponent oxidation, multiorganelle dysfunction, DNA breakage, and eventual cell death.^9,10^ Recent studies have highlighted the potential for antioxidants to protect RPE cells from oxidative stress as a therapeutic strategy against AMD. For example, antioxidant multivitamins and zinc supplements have been shown to be beneficial in animal models of AMD, although they cannot halt the progression of the disease.^11^

Cell-based “phenotypic” high-throughput screening (HTS) is a powerful unbiased tool to identify gene targets or small-molecule compounds exerting the desired effect.^12^ In a disease-associated HTS study, thousands of chemicals can be screened and analyzed in a short time period with viable repeatability. The high efficacy and convenience of HTS and high content analysis (HCA) have enabled the development of many new drugs or drug candidates identified in the last decade, such as modulators of transthyretin to treat transthyretin amyloidosis and the well-known BCR-ABL kinase inhibitor Gleevec to treat chronic myelogenous leukemia.^13,14^ The results of HTS screening typically identify new small molecules with a single protein or gene target in the cell, which makes it easier for further mechanistic studies to be conducted. Other advantages of the small-molecule model include convenient administration, flexible dosage determination, and low cost. However, to date, a comprehensive and systematic screening method to identify targets and compounds that can protect RPE cells against oxidative stress and that are effective in vivo has not been reported.

Herein, we report the development of a high-content, imaging-based assay that was used to systematically screen 814 protective compounds. We identified D609, a compound that protects RPE cells from sodium iodate (SI)-induced oxidative cell death in a dose- and time-dependent manner in both the ARPE-19 cell line and primary RPE cells. The antioxidative and mitochondrial protective ability of the selected candidate was evaluated. Then, the underlying antioxidative mechanism was explored. Finally, the in vivo efficacy of the selected candidate was evaluated.

## Results

### Identification of compounds that promote RPE cell survival

Taking advantage of the scalable and highly homogeneous RPE cell line ARPE-19, we aimed to develop a 384-well format HTS assay to identify lead chemicals that can protect RPE cells from oxidative damage and serve as potential therapeutics for AMD. We chose a standard model of inducing oxidative stress and cell death—NaIO_3_ (sodium iodate, SI) stimulation—and applied it to ARPE-19 cells. Various plating densities, as well as the concentration and duration of the SI treatment, were optimized for a 384-well HTS format, and we achieved ~90% cell death in 24 h (more than 20-fold higher than that in the untreated control cells).

**Fig. 1:**
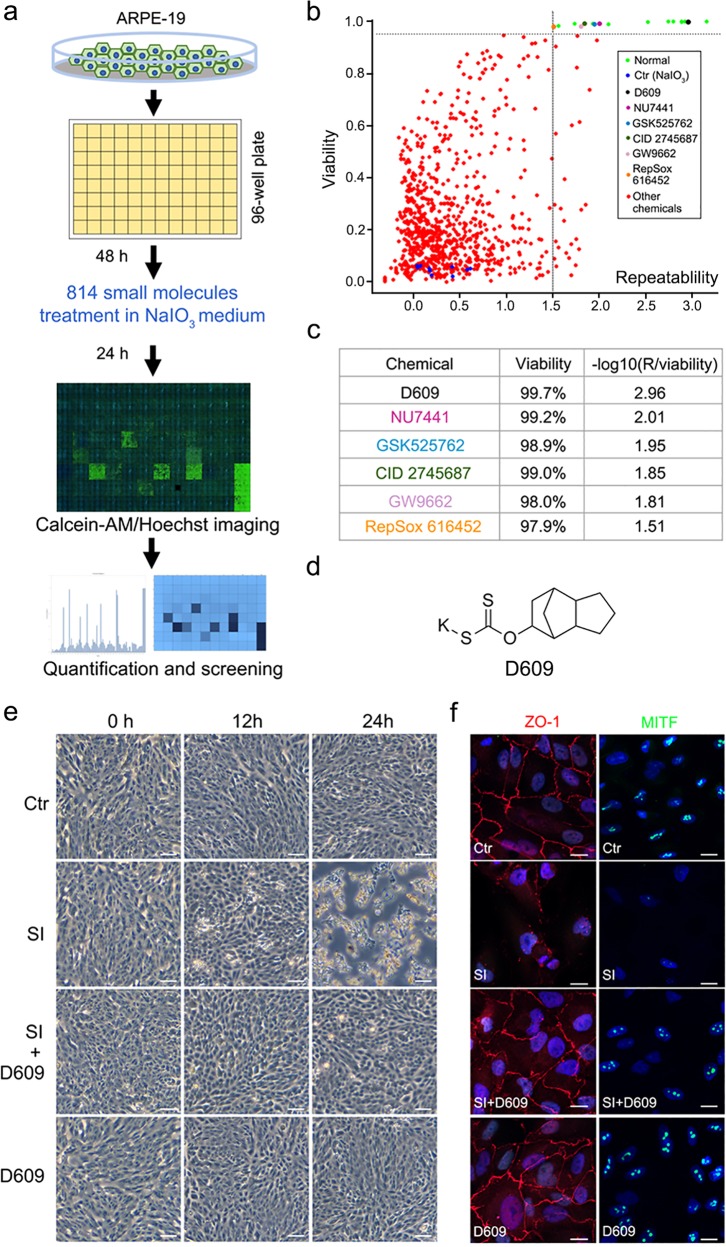
High-content screening of oxidative stress inhibitors in ARPE-19 cells. **a** The workflow of the high-content screening method. **b** Analysis of the effects on cell viability of each compound by calcein-AM/Hoechst imaging in ARPE-19 cells. *R* is the value of the distance between the two times of analysis of the viability. **c** The list of chemical candidates in the library that can inhibit SI-induced cell death in ARPE cells. **d** Chemical structure of D609. **e** Phase-contrast images of the ftRPE cells treated with D609 (10 μM), SI (10 μM), or a combination at 0, 12 or 24 h. **f** Immunofluorescence imaging of ZO-1 and MITF in the ftPRE cells treated with D609, SI, or a combination for 18 h. Scale bar: 100 μm (**e**), 20 μm (**f**). *n* = 3

The workflow of the screening is summarized in Fig. 1a. A collection of 814 protective small-molecule compounds was screened in two independent experiments (Fig. 1b). Compounds were selected from a larger collection based on a combination of criteria including a balanced number of external/internal (known and propriety) compounds, diversity of the annotated targets, and known targets associated with cell protection in our previous experiments.^15^ The primary screen identified 59 compounds with an average survival response greater than 10-fold, although most chemicals showed repeatable cell viability results (Fig. 1). We identified six small molecules that could robustly and reproducibly protect the RPE cells from SI-induced cell death, resulting in strongly enhanced cell viability from <10% to as much as 97% (Fig. 1b, c, and Fig. S1). The detailed post-treatment images of the six chemicals are shown in the Supplementary Material Section (Fig. S1b).

After comparing the effectiveness and post-treatment cellular morphology following addition of all the promising compounds from the primary screening, we identified the best compound as tricyclodecan-9-yl-xanthogenate (D609), a xanthate derivative that consistently showed the most effective protection of cell survival (chemical structure in Fig. 1d). D609 not only prevented the SI-induced cell death at the highest ratio but also maintained normal cellular morphology (Fig. 1c and S1b). Therefore, we selected D609 for further study. The applied concentration of D609 was 10 μM, as determined by a dose-dependent CCK8 assay in the ARPE-19 cells (Fig. S1c), which showed optimized cell protection with a lower dosage.

To further clarify the antioxidative effect of D609 in the primary cells, which are more similar to an in vivo scenario, we evaluated D609 in human fetal RPE cells (ftRPE) and adult human RPE (hRPE) cells. The grouping setup was as follows: the control group, the D609-treated group as the negative control group, the SI-treated group as the oxidative damage group, and the D609-SI cotreatment group as the rescue group. Time-series phase-contrast brightfield imaging confirmed the protective function of D609. Some hRPE and ftRPE cells died after 12 h of SI treatment, and cell death was exacerbated when the treatment time reached 18 and 24 h, respectively. In the SI-D609 cotreatment group, the cell morphology was similar to that of the control group at each time point (Fig. 1e, S1d and S1e), implying a broad function in the RPE lineages.

ZO-1 and MITF are well-defined markers of RPE cells^16^ that are located in the cell membrane and nucleus, respectively. The expression of these two markers was identified in the ftRPE cells by immunostaining. Both markers disappeared during the SI-induced cell damage process, which indicates either the loss of the RPE character or the collapse of the whole-cell structure during oxidative damage. Interestingly, D609 helped to maintain the expression and subcellular localization of both ZO-1 and MITF (Fig. 1f).

### D609 inhibited the SI-induced ftRPE necrotic cell death

**Fig. 2:**
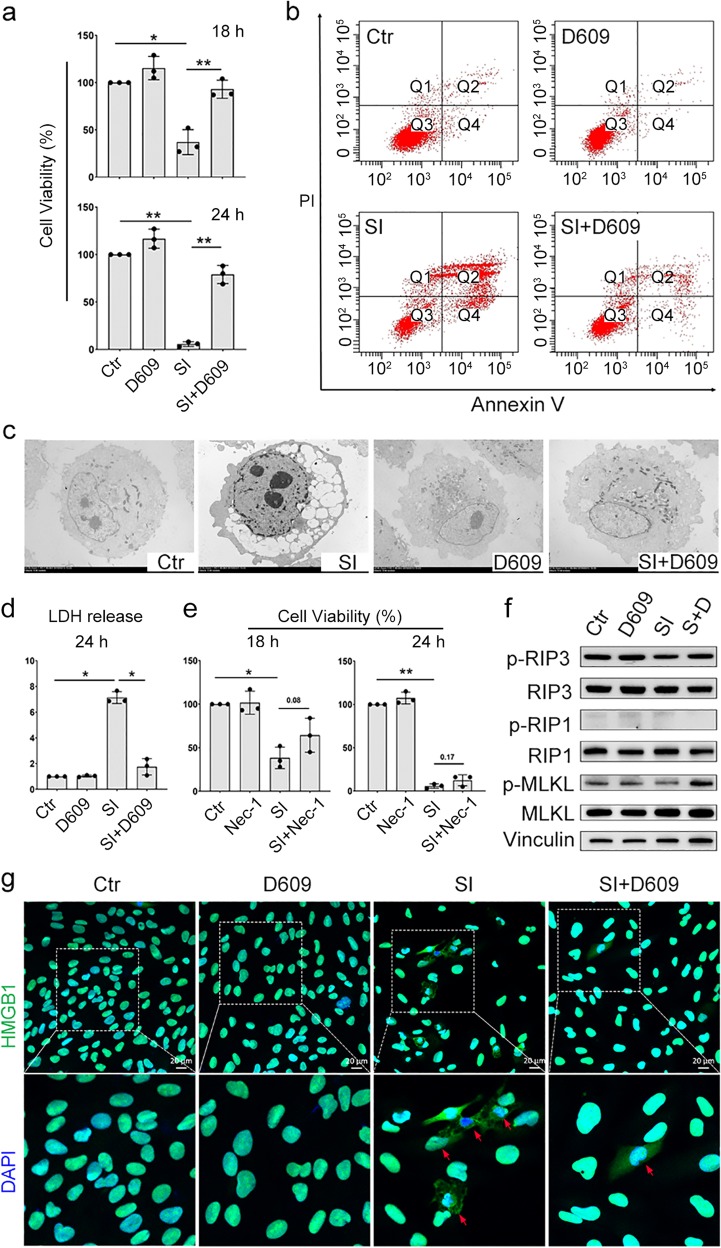
D609 inhibited SI-induced ftRPE cell death. **a** ftRPE cells treated with D609 (10 μM), SI (10 μM), or combination for 18 h or 24 h, and cell viability tested by CCK8-kit. Data are shown as the mean ± SD. **b** Cell death analyzed by flow cytometry using Annexin V and PI double-staining. ftRPE cells treated with D609 and/or SI for 18 h. Quadrant 1 and 2 (PI-positive) represent dead cells, and quadrant 2 and 4 represent apoptotic cells. **c** Transmission electron microscopy images of the ftRPE cells treated with D609 and/or SI for 18 h. **d** LDH release of the ftRPE cells treated with D609 (10 μM), SI (10 μM), or a combination for 24 h. Data are shown as the mean ± SD. **e** Cell viability (CCK8) of the ftRPE cells treated with 30 μM of the necroptosis inhibitor necrostatin-1 (Nec-1) and/or SI (10 μM) for 18 or 24 h. Data are shown as the mean ± SD. **f** Western blot analysis of phospho-RIP3, RIP3, phospho-RIP1, RIP1, phospho-MLKL, and MLKL in the ftPRE cells treated with D609 and/or SI for 18 h; vinculin was used as the loading control. **g** Expression and location of HMGB1 in the ftPRE cells treated with D609 and/or SI for 18 h by immunofluorescence imaging. *p < 0.05, **p < 0.01. *n* = 3

A series of cytotoxic analyses were carried out to further clarify the D609 antagonism of SI in the ftRPE cells. First, the cytoprotective ability of D609 was verified by a CCK8 assay in the ftRPE cells under severe oxidative stress. After 18–24 h of SI treatment (10 μM), the CCK8 results indicated that the viability of the ftRPE cells decreased dramatically to under 20%, but the SI-D609 cotreatment group had a value higher than 95% (Fig. 2a).

Previous in vitro AMD studies have reported the existence of apoptosis and necrosis in oxidant-induced RPE injury.^9,17^ In this study, to reveal the exact effect of D609 in SI-induced RPE injury, we performed propidium iodide (PI)/annexin V flow cytometry to elucidate the cell death types following treatment of each group. These results showed that the PI-positive cells, representing necrotic cells and late-stage apoptotic cells, were strongly increased after SI treatment, and this treatment also slightly increased the early apoptotic cell population (PI-/Annexin V+). However, both the necrotic and apoptotic cell populations decreased sharply in the SI-D609 cotreatment group (Fig. 2b), suggesting the potent antinecrotic and antiapoptotic effects of D609 in the ftRPE cells. Transmission electron microscopy (TEM) confirmed that necrosis was the predominant cell death form and that D609 could rescue this process in ftRPE cells. The TEM images showed that the majority of the SI-treated cells exhibited oncotic cell death, cytoplasmic blebbing, low electron density, and missing organelles, which are all indicators of necrosis (Fig. 2c). Necrosis of the ftRPE cells caused by SI and its inhibition by D609 were also detected by the lactate dehydrogenase (LDH) release assay using culture medium after 24 h of treatment (Fig. 2d).

Hanus et al. reported that RPE cells can undergo necroptosis in response to SI.^18^ In our study, the necroptosis inhibitor necrostatin-1 (Nec-1) slightly inhibited SI-induced ftRPE cell death after 18 or 24 h of cotreatment (Fig. 2e). The current methodology of necroptosis identification also includes relocation of the nuclear necroptosis-specific cytokine high-mobility group box-1 (HMGB1) and phosphorylation of the receptor interaction proteins 1 (RIP1)/receptor interaction proteins 3 (RIP3)/mixed lineage kinase domain like (MLKL) signaling cascade.^19^ Our immunostaining results showed that SI treatment could cause the leakage of HMGB-1 from the nucleus to the cytoplasm, and D609 cotreatment effectively prevented this leakage (Fig. 2g). However, the immunoblotting results showed no obvious alteration of RIP1/RIP3/MLKL signaling phosphorylation in either the SI-treated or SI-D609-cotreated ftRPE cells (Fig. 2f). Notably, necroptosis, which is a programmed type of necrotic cell death, belongs but is not equal to necrosis. Although flow cytometry analysis suggests that necrosis is the predominant form of cell death induced by SI, the fact that RIP1/RIP3/MLKL signaling is not changed and that Nec-1 has no significant rescue effect demonstrated that programmed necroptosis is not obviously stimulated. Therefore, among the types of cell death exhibited after oxidative stress, RIP1/RIP3/MLKL-independent necrosis prevails as the major form in vitro.

In addition to SI, tert-butyl hydroperoxide (tBH) was used as an alternative oxidizing reagent to test the specificity of D609 in ARPE-19 cells. D609 also showed a potent cytoprotective effect in the tBH-induced acute oxidative cell death (6 h) in ARPE-19 cells, as demonstrated by CCK8, LDH, PI/Annexin V flow cytometry and HMGB-1 immunostaining assays (Fig. S2). Nec-1 also had no obvious protective effect on the ARPE-19 cells treated with tBH, which indicates that tBH may not induce necroptosis in RPE cells (Fig. S2d).

### D609 inhibited the oxidative and mitochondrial damage in ftRPE cells

**Fig. 3:**
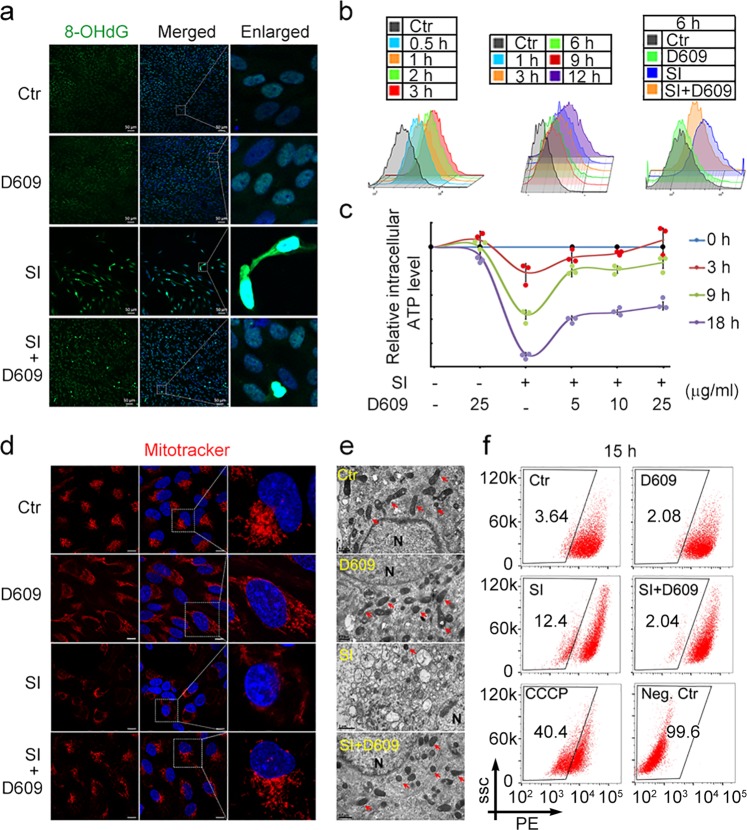
D609 inhibited SI-triggered oxidative damage and mitochondrial dysfunction in ftRPE cells. **a** DNA oxidative damage determined by 8-OHDG immunofluorescence imaging in the ftRPE cells treated with D609 (10 μM) and/or SI (10 μM) for 18 h. **b** ROS production level of the ftRPE cells treated with 0–12 h of SI (10 μM) and cotreatment with D609 (10 μM) for 6 h measured by flow cytometry. **c** Cellular ATP levels in the ftRPE cells treated with D609 and or SI for 18 h. Data are shown as the mean ± SD. **d** MitoTracker imaging represents the mitochondrial mass loss after 18 h of SI (10 µM) treatment in the ftRPE cells, while cotreatment of D609 (10 μM) rescued the mitochondrial loss. **e** Transmission electron microscopy images of the ftRPE mitochondria in the cells treated with D609 and/or SI for 18 h. **f** Mitochondrial transmembrane potential (MTMP) measured in the ftRPE cells treated with D609 (10 μM) and/or SI (10 μM) for 15 h by flow cytometry of TMRE staining. Carbonyl cyanide 3-chlorophenylhydrazone (CCCP) was used as a positive control to decrease MTMP. The no staining group was used as a negative control (Neg. Ctr). *n* = 3

To identify whether D609 inhibits oxidative stress-induced cell death in RPE cells via its previously reported antioxidative mechanism in cells,^20,21^ we examined the DNA oxidative damage indicators 8-hydroxy-2′-deoxyguanosine (8-OHdG) and reactive oxygen species (ROS), which are classic oxidative stress biomarkers.^22,23^ The 8-OHdGimmunostaining results showed that D609 could efficiently decrease the 8-OHdG-positive ftRPE cell number, which was increased by SI (Fig. 3a). Flow cytometry using the DCFH-DA probe indicated that the ROS levels were upregulated in the ftRPE cells treated with SI for different lengths of time (Fig. 3b). However, the ROS level was significantly decreased in the SI-D609 cotreatment group in the early stage (6 h) (Fig. 3b), which theoretically prevented further oxidative signal transduction. Cellular ATP depletion is another indicator that the cells are under oxidative stress.^24^ We also found that D609 could recover the SI-induced cellular ATP depletion in the ftRPE cells in a time- and dose-dependent manner (Fig. 3c).

Cellular ATP depletion is not only an indicator of oxidative stress but also a biomarker for mitochondrial dysfunction. In addition to oxidative stress, the health status of RPE mitochondria was shown to be critical in AMD pathology. The architecture, function, and mass of mitochondria all closely correlate with the severity of AMD.^25,26^ MitoTracker staining in the ftRPE cells showed that the mitochondrial mass dramatically decreased after SI induction (Fig. 3d), and TEM imaging also showed that the mitochondrial number was decreased after SI induction (Fig. 3e). Cotreatment with D609 prevented the mitochondrial loss to maintain the RPE cells in a healthy state (Fig. 3d, f). The mitochondrial membrane potential (MMP) was also examined in the ftRPE cells by the MMP probe JC-1, and the results indicated the unhealthy polarized status of the mitochondria following SI treatment and the expected protective effect of D609 (Fig. 3f). The inhibitory effect of D609 on ROS overproduction and mitochondrial dysfunction was also verified in ARPE-19 cells and after tBH treatment (Fig. S3a-c).

D609 was shown to initiate autophagy in human umbilical vein endothelial cells (HUVECs) and hippocampal neurons.^27,28^ Through coimmunostaining of the autophagosome indicator LC3 and MitoTracker, we also found that many mitochondria colocalized with autophagosomes when the ftRPE cells were treated with SI, indicating that the disappearance of the mitochondria was caused by mitophagy, the classic degradation pathway of mitochondria (Fig. S3d). In the presence of the specific autophagy inhibitor SPT1, D609 showed a compromised ability to protect the ftRPE cells from SI-induced cell death, indicating the prosurvival role of autophagy in RPE cells (Fig. S3e).

Taken together, all the oxidative and mitochondria-related results indicated that D609 has potent antioxidative effects that could decrease excessive oxidative stress signals and protect the homeostasis of the mitochondria in RPE cells.

### D609 inhibits oxidative damage by upregulating metallothionein

D609 is a selective competitive inhibitor of phosphatidylcholine-specific phospholipase C (PC-PLC).^29,30^ PC-PLC can catabolize phosphatidylcholine to generate phosphocholine and diacylglycerol (DAG), which is an important second messenger. To determine whether the inhibition of PC-PLC by D609 contributes to its antioxidative effect in RPE cells, we examined the PC-PLC activity in the ftRPE cells treated with SI and D609. The results showed that D609 inhibited the PC-PLC activity in the ftRPE cells after 9 h and 18 h of treatment, but the PC-PLC activity in the SI and SI-D609 groups also decreased to a comparable extent at the same time points (Fig. S4a). This similar trend of enzyme activity theoretically ruled out PC-PLC participation in the antioxidative effect of D609.

**Fig. 4:**
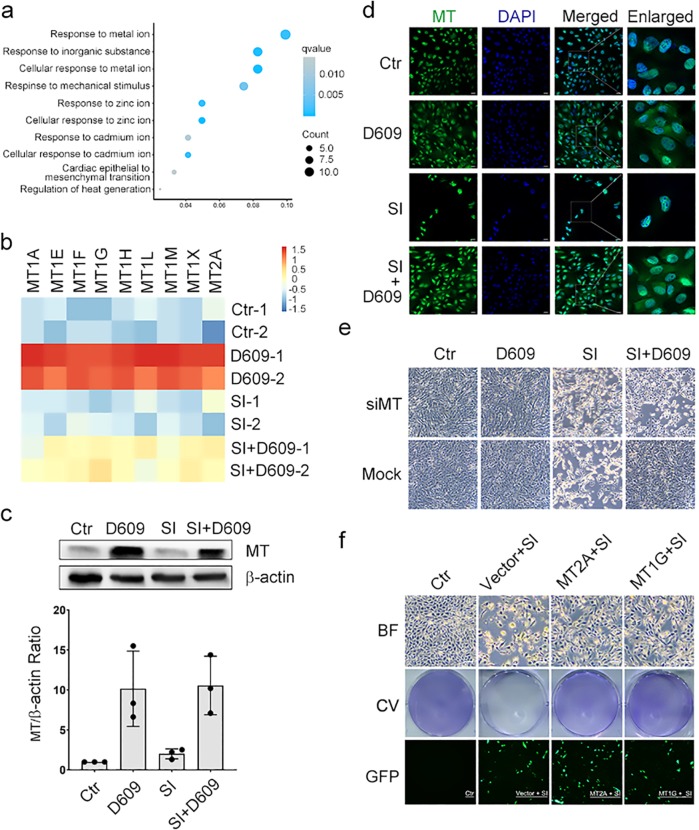
D609 inhibits SI-induced oxidative cell damage by upregulating the MT protein family. **a** GO analysis of the upregulated genes between the SI-cotreated D609 group vs. the SI-treated group from ftRPE RNA-Seq data. **b** Heatmaps of the MT family genes at the transcriptional level. **c** Left panel: Western blot analysis of MT in the ftPRE cells treated with D609 and/or SI for 12 h; β-actin was used as the loading control. Right panel: quantification of the western blots. Data are shown as the mean ± SD. **d** Immunostaining images of MT in the ftRPE cells treated with D609 (10 μM) and/or SI (10 μM) at 18 h. (**e**) Phase-contrast images of the ftRPE cells after RNA interference of MT and treatment with D609 (10 μM) and/or SI (10 μM) for 18 h; scrambled siRNA was used in the Mock group. **f** Phase-contrast bright field (BF), crystal violet (CV) staining and GFP images after D609 (10 μM) and/or SI (10 μM) treatment in the MT2A and MT1G-overexpressing ARPE-19 cells. *p < 0.05. *n* = 3

RNA sequencing (RNA-Seq) was performed to further investigate the antioxidative mechanism of D609 (Fig. S4b). In gene ontology (GO) analysis of the differentially expressed genes, the top 10 categories of the enrichment results were primarily associated with metal responses such as zinc and cadmium in the SI versus SI-D609 cotreatment cohorts (Fig. 4a). From the detailed metal response gene list, we determined that metallothionein (MT) family genes showed the strongest regulation in response to D609; these proteins (41), including MT1A, MT1E, MT1F, MT1G, MT1H, MT1L, MT1M, MT1X, and MT2A, have potent antioxidant properties. Differential gene expression analysis also showed that cotreatment with D609 significantly altered the expression of the MT genes compared to the SI-only treatment (Fig. 4b).Among all the increased MT genes following D609 treatment (Fig. 4b), MT1E, MT1G, MT1X, and MT2A were dramatically upregulated in the presence and absence of SI induction in the ftRPE cells (Fig. S4c, d). In addition, the protein level of MTs was translationally increased by D609 (Fig. 4c), and MT expression could be observed in the ftRPE cells (Fig. 4d). Collectively, D609 effectively upregulated the expression of the potent MT antioxidant family. We also examined the expression of other major antioxidants by RNA-Seq analysis to verify their involvement; however, no other antioxidants in the GPX or SOD family were regulated by D609, as demonstrated in Fig. S4e.

To study the role of MT in the inhibition of cell death processes, we designed an siRNA that targeted the conserved coding sequence (CDS) of MT mRNAs (Fig. S4f, G, and H). The siRNA decreased the expression of MT genes with high efficiency (Fig. S4i). Upon knockdown of MT, D609 failed to protect the ftRPE cells from SI treatment for 18 h. Moreover, the downregulated MT was associated with accelerated cell death compared to that in the negative control group (Fig. 4e). Upon knockdown of MT, the ROS level did not decrease, and the mitochondrial membrane did not show improvements after SI-D609 cotreatment, as found in normal RPE cells (Fig. S4j, k). Knockdown of MT by RNAi also efficiently inhibited the cytoprotective function of D609 in the tBH-treated ARPE-19 cells (Fig. S2e, f).

To further elucidate the role of MT, we generated overexpression plasmids (GFP-labeled). Upon increased expression of MT2A and MT1G, ARPE-19 cells showed relatively higher survival post-SI treatment, as demonstrated by bright field imaging and whole-mount crystal violet staining (Figs. S4l, f). Notably, the cell protective effect of MT2A and MT1G overexpression was not as strong as that of the D609 treatment, which suggests that individual MTs could only partially facilitate the effects of D609. Hence, the antioxidative and cytoprotective effects of D609 were not achieved through its own reducibility but were instead primarily dependent on its dramatic capability to upregulate MT.

### D609 rescued oxidative RPE damage in a dry AMD mouse model

**Fig. 5:**
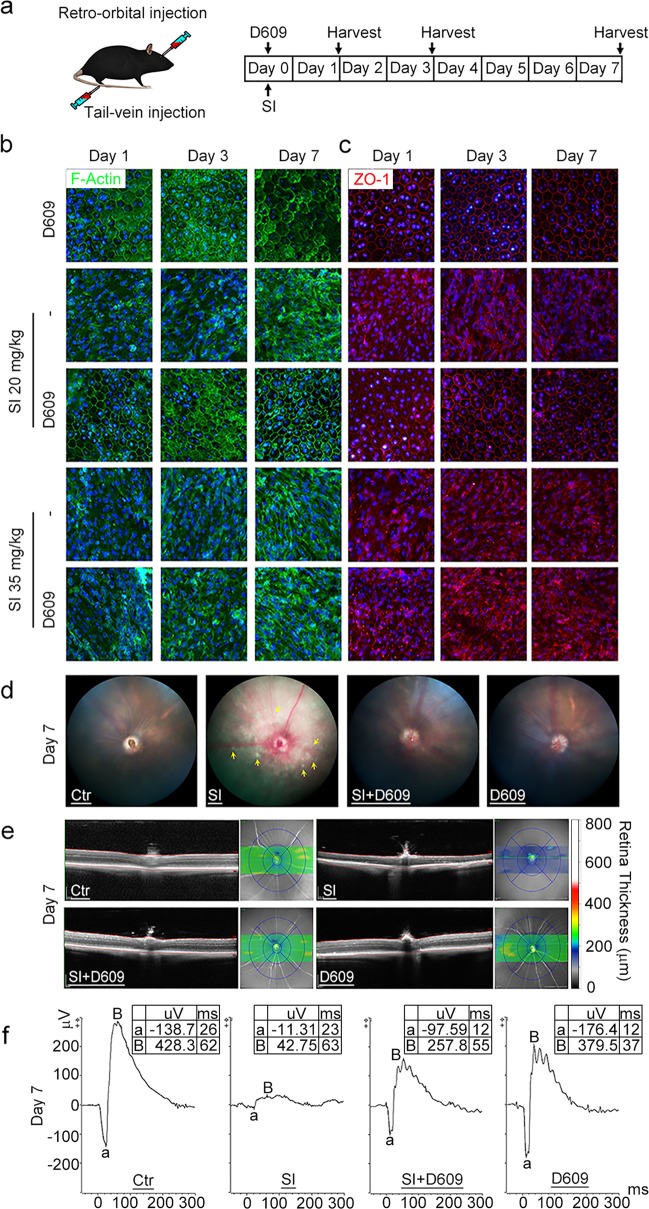
D609 could rescue the RPE cell damage caused by SI injection in mice. **a** Schematic diagram of the timepoint design for the AMD mouse model via SI tail-vein injection. D609 (25 mg/kg) and SI (20 mg/kg or 35 mg/kg) were injected simultaneously, and the mouse retinas were collected at day 1, day 3 and day 7 post-injection. **b** F-actin structure of the day 1, day 3, and day 7 post-injected mouse retina by phalloidin staining. **c** Expression of ZO-1 in the treated mouse retinas by immunofluorescence imaging. **d** Fundus photograph of the mice taken at day 7 after injection with D609 (25 mg/kg) and/or SI (20 mg/kg). **e** Representative mouse OCT images of the central retina and retinal thickness at day 7 post-injection with D609 (25 mg/kg) and/or SI (20 mg/kg). **d** Mouse mesopic ERG responses (3 cd.s.m^−2^) recorded at day 7 post-injection with D609 (25 mg/kg) and/or SI (20 mg/kg); *n* = 5

Given the significant cytoprotective effect of D609, we further tested its potential as a therapeutic for AMD in vivo. Mice with tail-vein SI injections were used as the AMD model, and D609 was injected retro-orbitally. The schematic administrative procedure is shown in Fig. 5a. For the injection dose of D609, the order of magnitude was determined according to previous reports,^20,31,32^ and the optimal dose (25 mg/kg) was determined by preliminary injections with a concentration gradient of 5, 25, and 50 mg/kg. The dose of 25 mg/kg had a viable antioxidative ability with no RPE toxicity, as shown in the 50 mg/kg injection group (Fig. S5a). Dissection and H&E staining showed no obvious toxicity in the major organs of the 25 mg/kg D609-injected mice, including the heart, liver, and kidneys (Fig. S5b), which indicated the safety of D609. After collection of the mouse retinas at postinjection days 1, 3 and 7, RPE flatmount staining of ZO-1 and F-actin was performed. The SI-injected retinas with both low (20 mg/kg) and high (35 mg/kg) dosages showed severe dysmorphology of the RPE layer from postinjection day 1 to day 7, while the retinas from the control group (D609 injection) or the coinjected group (low SI dosage) showed perfect polygonal RPE cells (Fig. 5b, c). However, D609 failed to prevent retinal damage caused by the high dose of SI (Fig. 5b, c).

To assess the geographic atrophy phenotype of the retina, we performed a fundoscopic examination in the SI- and D609-injected live mice at 7 days postinjection. The fundus image of the SI-injected mice appeared to be brighter than that of the control group and showed mottling, as indicated by the yellow arrow in Fig. 5d. The SI-D609 coinjected retinas appeared similar to those in the control group with no sign of geographic atrophy (Fig. 5d). To further evaluate the physiological function of the retinas following injection, we performed optical coherence tomography (OCT) in living mice. OCT images demonstrated that SI injection could cause thinning of the whole retina layer, which was indicated by the thickness heatmap (Fig. 5e). In addition to thickness, loss of retinal lamination and hyperreflective opacities were also observed in SI-injected retinas. All phenotypes associated with SI are indicators of severe retinal damage and were not present in the SI-D609 coinjected mouse retinas (Fig. 5e).

After 7 days of SI injection, the scotopic response of the retinas was examined by electroretinography (ERG). The amplitudes of the A-waves and B-waves were all dramatically reduced after SI injection, demonstrating the dysfunction of the photoreceptors and bipolar cells by oxidative damage (Fig. 5f). However, the SI-D609-coinjected mice presented a relatively good response to light stimuli in both the amplitudes of the A-waves and B-waves (Fig. 5f). The protective effect of D609 observed by OCT and ERG was prolonged to 30 days postinjection (Fig. S5b). Together with the OCT results, the ERG observations indicate that D609 could effectively antagonize oxidative stress and protect the physiological function of the retinas in vivo.

## Discussion

Mounting evidence has shown that the dysfunction and loss of RPE cells induced by oxidative stress is critical to the pathophysiology of dry AMD.^33^ Herein, using HTS combined with image-based HCA, we identified D609 as a small antioxidative molecule from 814 candidates and showed that it can efficiently inhibit oxidative damage-induced RPE cell death both in vitro and in vivo. D609 extensively inhibited excessive ROS and protected mitochondria in the RPE cells under oxidative damage. This therapeutic effect of D609 in RPE depends on its strong ability to upregulate the expression of MT, a family of antioxidative proteins. Collectively, the potent antioxidative and cytoprotective effects make D609 a possible candidate for dry AMD treatment.

SI treatment was used to establish experimental oxidative damage models for drug screening and mechanistic exploration in RPE cells. Although the rapid induction of AMD by SI more closely resembles an acute disease model rather than the long aging process of AMD, SI induction could efficiently initiate most AMD phenotypes and is broadly used in related studies.^34,35^ Consistent with other studies,^17,18^ both the apoptotic and necrotic types of cell death were identified under SI treatment, with the necrotic phenotype more dominant, as seen in the TEM and LDH results. In particular, we found that the necroptosis inhibitor Nec-1 partially rescued SI-induced cell death but did not have an obvious impact on RIP1/RIP3/MLKL signaling, indicating the limited involvement of programmed necrotic cell death.

A sufficient autophagy level is believed to be critical for the survival of most cell types, and impaired autophagy is associated with RPE degeneration and increased susceptibility to oxidative stress.^36^ Indeed, we noted that autophagy may participate in protection of the RPE due to the antagonizing effect of the autophagy inhibitor SPT-1 on D609. However, considering the nonsignificant increase in autophagy induced by D609 and the acute death process caused by SI, autophagy appears to have limited involvement and effectiveness in the cytoprotective process within RPE cells. Autophagy may not contribute to the cytoprotective mechanism of D609 in the current study, but for the long-term management of AMD, maintaining a relatively high level of autophagy in the RPE could be a valid precautionary method. In addition to the direct involvement in RPE protection, D609 injection may also protect neuroretinas from SI injection-induced injury, which further contributed to its potential effect on dry AMD.

D609 has been shown to be an antioxidant in different cell types;^21,31^ however, its antioxidative mechanism has not been clearly elucidated. In this study, we found that D609 dramatically increased the expression of MT, regardless of whether the RPE cells were under oxidative stress. The cytoprotective effect of D609 was significantly decreased when MT expression was silenced by siRNA interference, indicating the essential role of MT in the antioxidative effects of D609. According to our results, although D609 can partially eliminate ROS and counteract oxidative stress,^20,31,32^ in RPE cells, it achieves its antioxidant effects primarily through elevating the expression of MT. The MT family is well known for its ability to eliminate oxidative risk factors, such as scavenging ROS via the thiol groups in the cysteine residues, and its antioxidative activity is more potent than that of most known antioxidants.^37–39^ The antioxidative and heavy metal binding effects endow MT with a formidable cytoprotective capability in different tissues, and overexpression of MT could be beneficial for diabetic cardiomyopathy and islet transplantation.^40,41^ MT1G, MT1X, and MT2A showed relatively high expression in untreated RPE cells and were significantly upregulated by D609; thus, we hypothesized that they might be the pioneer members to provide antioxidative activity. Further studies are needed to identify the major members of the MT family in RPE cells.

Cell transplantation using embryonic stem cells (ESCs) or induced pluripotent stem cell (iPSC)-derived RPE cells is a promising therapy for AMD.^42–44^ ESC/iPSC-derived RPE cells are largely applied to AMD patients in the most advanced stage. Therefore, seeking novel therapies or drugs that can rescue RPE cells from death will strongly benefit patients in the progressive stages. Small molecule-based therapeutics have irreplaceable advantages in terms of cost and administration compared with emerging cell therapies. D609 is also water soluble and has not been shown to induce damage to major organs based on our preliminary data. Although future studies are needed to determine the mechanism employed by D609 in MT elevation, we believe our findings broaden the possible application of this small molecule for a series of oxidative stress-related diseases, including but not limited to AMD.

## Materials and methods

### Compound library screening

Briefly, subconfluent ARPE-19 cells were simultaneously treated with SI alone or with the library chemicals for 24 h, and cell survival was determined by a standard calcein-AM/Hoechst cytotoxic assay and quantified by high-content imaging analyses. These compounds consisted of ~400 well-characterized inhibitors against many kinases, phosphatases, and other signaling receptors, as well as ~200 epigenetic modifiers, ~100 metabolic modulators, and ~150 cell death and stress alleviators. Small molecules in these libraries were purchased from Sigma, Tocris Bioscience, Merck, Cayman, Selleckchem, and Stemgent.

### Cell culture

The immortal human RPE cell line ARPE19 was obtained from the American Type Culture Collection (ATCC, CRL-2302) and cultivated in an appropriate medium (1:1 of Dulbecco’s modified Eagle’s medium/F12 with 10% fetal bovine serum, supplemented with 1% penicillin/streptomycin). Primary fetal and adult human RPE cells were prepared as described previously.^45^ In brief, primary RPE cells were harvested from human fetal eyes. The adult human RPE donors were 40–50 years old. After the eye cups were incubated in dispase solution for 40–60 min at 37 °C, the retina was cut away from the optic nerve, and the RPE layer was nicely stretched. Fetal RPE and adult human RPE cells were cultivated in an appropriate medium (MEM-alpha modified medium, supplemented with 10% fetal bovine serum, N1 supplement 1:100, GlutaMax/ penicillin-streptomycin 1:100, and nonessential amino acid solution 1:100, hydrocortisone (20 μg/L), taurine (250 mg/L), and triiodothyronine (0.013 μg/L)). The cells were grown at 37 °C in a humidified atmosphere of 5% CO_2_.

### Animal model and treatment

C57BL/6 mice (male, 6–7-week-old, weight range: 18–23 g) were purchased from Guangdong Laboratory Animal Center (Guangzhou, China) and maintained in the animal facility of the Zhongshan Ophthalmic Center at Sun Yat-sen University (Guangzhou, China). Sodium iodate (Sigma, #71702) and D609 (R&D Systems, #1437) were diluted in sterile phosphate-buffered saline (PBS) to concentrations of 2 mg/mL and 20 mg/mL and stored at 4 °C. SI was injected via the tail vein. D609 was injected via the orbital venous plexus. The control mice were injected with the same volumes of PBS.

### Calcein-AM/Hoechst staining

Cells were incubated with Calcein-AM dye by using the Cellstain-Double Staining Kit (Dojindo, #C542) and Hoechst (Life, #H3570) for 15 min at 37 °C. Living cells stained green were observed using the Operetta CLS High-Content Analysis System (Perkin Elmer, USA).

### CCK8 assay

A CCK8 assay kit was obtained from Dojindo (Dojindo, #CK04). Briefly, cells were seeded in 96-well plates and exposed to conditioned medium. Then, 100 µL of a mixture of culture medium and CCK8 solution was added to each well of the plate, and the plate was then incubated for 1 h (at 37 °C and 5% CO_2_). The absorbance at 450 nm was measured by using a microplate reader (BioTek Instruments, USA).

### Cell apoptosis assay

After culture in conditioned medium, cells were rinsed and collected by centrifugation for Annexin V-FITC/PI staining (BD Biosciences, #556547). Each pellet was resuspended in 500 μL of binding buffer. Then, 5 μL of FITC and 5 μL of PI were added to each well, and the cells were incubated for 15 min at 37 °C. The apoptotic ratios were then determined by flow cytometry (BD LSRFortessa™ cell analyzer, USA).

### Crystal violet staining

Cells were seeded in six-well plates and exposed to conditioned medium until the cell density reached 80% confluence. Before evaluation, the cells were washed with PBS and fixed with 4% paraformaldehyde for 15 min at room temperature. Then, the plate was washed with tap water and stained with 500 µL of 0.5% crystal violet staining solution (Beyotime, #C0121) for 45 min at room temperature. The plate was then washed with tap water three times and air-dried before scanning.

### ROS activity measurement

Cellular ROS production was measured using a 2′,7′-dichlorofluorescein diacetate (DCFH-DA) assay kit (Abcam, #ab113851) as described in a previous report.^46^ Briefly, cells were grown in conditioned medium in six-well plates. When confluent, the cells in the six-well plates were rinsed and incubated with 25 mM DCFH-DA at 37 °C for 30 min. The fluorescence intensity was examined by flow cytometry.

### Mitochondrial membrane potential assay

MMP was measured with a Mitochondrial Membrane Potential Assay Kit (CST, #13296S). Briefly, after culture in conditioned medium, cells were rinsed and washed 3 times with phosphate-buffered saline (PBS) and incubated with 50 nM TMRE for 20 min at 37 °C. At the end of the incubation, the cells were washed in PBS and then analyzed by flow cytometry at an excitation wavelength of 488 nm and an emission wavelength of 575 nm.

### Lactate dehydrogenase assay

LDH release was analyzed using the Pierce™ LDH Cytotoxicity Assay Kit (Thermo Scientific, #C20300) as described previously.^47^ Briefly, aliquots of cell-free supernatant were collected in a 96-well plate in triplicate, and LDH reaction mixture was added to each well. After a 30-min incubation at room temperature, stop solution was added to each well, and the absorbance at 490 and 680 nm of each well was determined by a microplate reader.

### ATP assay

The CellTiter-Glo Luminescent Cell Viability Assay (Promega, #G7570) was used per the manufacturer’s instructions. Briefly, after cells were exposed to conditioned medium in 96-well plates, 100 µL of CellTiter-Glo® Substrate reagent medium was added to each well. The contents were mixed for 2 min on an orbital shaker to induce cell lysis. Then, the plates were incubated at room temperature for 10 min to stabilize the luminescent signal, and the luminescence was detected using a microplate reader.

### MitoTracker staining

MitoTracker Red dye was prepared according to the manufacturer’s recommendation (CST, #9082S). Briefly, a 500 nM working solution in prewarmed culture medium was prepared just prior to staining the cultured cells. After incubation for 30 min at 37 °C, the cells were washed with PBS and fixed in 4% paraformaldehyde for further immunofluorescence procedures.

### PC-PLC activity assay

Relative PC-PLC activity was determined in whole-cell lysates using the Amplex™ Red Phosphatidylcholine-Specific Phospholipase C Assay Kit (Thermo Scientific, #A12218), as described by the manufacturer and adapted by Spadaro *et al*.^48^

### RNA interference

Cells were transfected using Lipofectamine™ RNAiMAX Transfection Reagent (Invitrogen, #13778150) with siRNAs targeting the conserved CDSs of all MT genes (#1: sense, 5′-CUGCUGAUGCCUAGCCAGUUGGUAA-3′; antisense, 5′-UUACCAACUGGCUAGGCAUCAGCAG-3′; #2: sense, 5′-CCUAGCCUUUGGGACCGCUUCUCGU-3′; antisense, 5′-ACGAGAAGCGGUCCCAAAGGCUAGG-3′) or Stealth siRNA (Invitrogen, USA) as a negative control treatment. Briefly, dried siRNA was dissolved in nuclease-free water to achieve a final concentration of 20 nM. Then, 2 µL of siRNA (20 nM) and 2 µL of Lipofectamine RNAiMAX were added to 100 µL of buffer. The mixtures were maintained at room temperature for 15 min to form complexes, and equal aliquots were then added to the culture plate. The medium was replaced after 24 h.

### MT2A and MT1G overexpression

The MT2A and MT1G-specific pcDNA3.1-GFP overexpression vector (pcDNA3.1-MT2A-GFP, pcDNA3.1-MT1G-GFP) and empty vector (pcDNA3.1-GFP) were self-designed and constructed by IGEbio (Guangzhou, China). ARPE-19 cells were seeded in six-well plates and cultured to 70–80% confluence. Then, the vectors were transfected into ARPE-19 cells using Lipofectamine 3000 (ThermoFisher, #L3000150) according to the manufacturer’s instructions. The cells were cultured for another 48 h for the following experiments.

### Immunofluorescence

Cells were cultured in sterile Millicell EZ-Slide eight-well glass plates (EMD Millipore, #PEZGS0816). Before evaluation, the cells were washed with PBS, fixed with 4% paraformaldehyde for 15 min, permeabilized with 0.1% Triton X-100 for 30 min, blocked with 3% BSA for 1 h 30 min, and then incubated overnight at 4 °C with the primary antibodies. After washing, the cells were incubated for 1 h with secondary antibody. Images were acquired using a confocal fluorescence microscope (Zeiss, LSM 800). The following antibodies were used for immunofluorescence: ZO1 (Invitrogen #40–2200), MITF (Invitrogen, #PA5–38294), F-actin (Phalloidin FITC, Yepsen Biotechnology, #40735ES75), Metallothionein (Abcam, #ab12228), HMGB1 (Abcam, #ab18256), LC3B (CST, #3868), 8-OHdG (Santa Cruz, #SC-393871), Alexa Fluor 488-labeled donkey anti-rabbit IgG (CST, #4412), Alexa Fluor 488-labeled donkey anti-mouse IgG (CST, #4408), Alexa Fluor 594-labeled donkey anti-mouse IgG (CST, #8890), and Alexa Fluor 594-labeled donkey anti-rabbit IgG (CST, #8889).

### RPE flatmount staining

The globes of the mice were cleaned of extraocular tissue under a dissecting microscope. An incision was made through the sclera 3 mm posterior to the limbus until the choroidal vessels were exposed. Tenotomy scissors were introduced through this incision into the suprachoroidal space, and the incision was extended 360° circumferentially. Four radial relaxing incisions were made in the sclera, and the neural retina was gently removed with the RPE facing up. The flatmount was then fixed in 4% paraformaldehyde in phosphate-buffered saline (PBS) for 15 min and permeabilized with 0.1% Triton X-100 for 30 min for further immunofluorescence procedures.

### Fundoscopic examination

Retinal morphology was examined in vivo by fundoscopy using a Micron III camera (Phoenix Research Laboratories, USA). Briefly, mice were anesthetized using chloral hydrate (400 mg/kg, i.p.). Pupils were dilated with 1% tropicamide and 2.5% phenylephrine. For maintenance of corneal moisture, Systane lubricant eye drops (Alcon, Inc., USA) were applied.

### Electroretinogram record

Full-field Electroretinograms (ERGs) were recorded in anesthetized mice after overnight dark adaptation as previously described.^49^ Briefly, the mice were anesthetized, and the pupils were dilated. Then, gold-wire active electrodes were applied on the corneas. Dark-adapted rod and mixed rod-cone responses were recorded on a Celeris instrument (Diagnosys, USA).

### Western blot analysis

Western blotting was performed as previously described.^50^ Briefly, the harvested cells were lysed using a Minute Total Protein Extraction Kit (Invent Biotechnologies, #SD001) containing 1% protease inhibitor cocktail (Sigma-Aldrich, #P8340). The protein samples were boiled for 10 min at 70 °C with loading buffer, electrophoresed on SDS-PAGE (Bio-Rad, #4561094) and then transferred to a PVDF membrane. Subsequently, the membranes were blocked with 10% (w/v) nonfat milk in TBST (TBS containing 0.1% Tween-20) for 1 h at room temperature. The blots were incubated overnight at 4°C with primary antibodies. After the membranes were washed with TBST, they were further incubated with secondary antibodies for 1 h at room temperature. The blots were visualized using an ECL kit according to the manufacturer’s instructions. The intensity of the bands was semiquantified using ImageJ software. The following antibodies were used for Western blotting: p-RIP3 (CST, #93654), RIP3 (CST, #13526), p-RIP1 (CST, #65746), RIP1 (CST, #3493), p-MLKL (CST, #91689), MLKL (CST, #14993), vinculin (CST, #13901), horseradish peroxidase (HRP)-conjugated anti-rabbit IgG (CST, #7076S), HRP-conjugated anti-mouse IgG (CST, #7072),and Metallothionein (Abcam, #ab12228).

### RNA isolation and gene expression analysis

Total RNA was extracted using the RNeasy Protect Mini Kit (Qiagen, #74124) and quantified with a spectrophotometer (NanoDrop ND-1000; Thermo Fisher Scientific, USA). cDNA synthesis was performed with 500 ng of total RNA using PrimeScript RT Master Mix (TaKaRa, #HRR036A). Gene expression was then measured by quantitative PCR (qPCR) with SYBR Green Supermix (Bio-Rad Laboratories, #1708880) using a QuantStudio 7 Flex system (Life Technologies, USA) and the appropriate primers. GAPDH was used as a reference gene. The sequences of the primers (5′−3′) used were as follows: MT1A-Forward: TCCTGCAAATGCAAAGAGT; MT1A-reverse: ACACTTGGCACAGCTCAT; MT1E-Forward: GCTTTCTGCCCTAAATTC; MT1E-reverse: CTCTGTTCTGAAACCATAG; MT1F-Forward: TTCCTGCAAGTGCAAAGAGT; MT1F-reverse: TTAGCCACAGCCCCAGAC;MT1H-Forward: TGGGAACTCCAGTCTCACCT; MT1H-reverse: TTCTTGCAGGAGGTGCATTT; MT1G-Forward: CCCTGCTCCCAAGTACAAAT; MT1G-reverse: GGAATGTAGCAAAGGGGTCA; MT1X-Forward: TTGGCTCCTGTGCCTGTG; MT1X-reverse: AGCAGCAGCTCTTCTTGC; MT2A-Forward: AGAAAAGCTGCTGCTCCTG; MT2A-reverse: GTCGCGTTCTTTACATCTG; GAPDH-Forward: TGTGGGCATCAATGGATTT;

GAPDH-reverse: ACACCATGTATTCCGGGTCAAT.

### Transmission electron microscopy

Cells were fixed overnight at 4 °C in 2.5% glutaraldehyde and 150 mM sodium cacodylate (pH 7.4). After postfixation in 1% OsO_4_ followed by uranyl acetate, the cells were dehydrated in ethanol and embedded in epoxy resin. Ultrathin sections were collected on formvar-coated grids and stained with uranyl acetate and lead citrate, and then, the samples were examined with a Hitachi HT7700 transmission electron microscope.

### RNA-Seq library preparation and data processing

RNA extraction was performed using RNeasy Plus (Qiagen, #74106) from the samples reported in this research. The RNA-Seq libraries were constructed by a TruSeq Stranded mRNA Library Prep kit (Illumina, #20020594) and sequenced on Illumina PE150 sequencers. Sequencing reads were mapped to hg19 using Star. TPM values were called using RSEM. For the time-series dataset, the genes having a TPM value of 0 across all time points were filtered out. Differential gene expression was defined at log_2_-fold change > 1. Differential expression was determined using DESeq2. Differentially expressed genes had a *p*-value < 0.01 as well as a log_2_-fold change > 1. The RNA-Seq data were from two batches of biological samples.

### Statistical analysis

All data are expressed as the mean ± SD. Statistical analyses were performed using GraphPad (GraphPad Software, USA). Student’s *t*-test was used to compare differences between groups.

### Study approval

All animal experiments complied with the Association for Research in Vision and Ophthalmology Statement for the Use of Animals in Ophthalmic and Vision Research (Guangzhou, China; approval ID: 2018–047) and were approved by the Institutional Review Board of Zhongshan Ophthalmic Center.

## Supplementary information

Data S1

Supplementary_Figures.

## Data Availability

The analyzed RNA-Seq transcriptome matrix is enclosed in the supplementary materials (Data S1). The original datasets are also available from the corresponding author upon request.
