# Supplementary material for: D609 protects retinal pigmented epithelium as a potential therapy for age-related macular degeneration
Source: Signal Transduct Target Ther. 2020 Mar 4;5:20. doi: 10.1038/s41392-020-0122-1 (PMC7054264; doi:10.1038/s41392-020-0122-1)
Supplement: Supplementary file 2 — Supplementary_Figures. [file 41392_2020_122_MOESM2_ESM.pdf]

Supplementary Materials for

D609 Protects Retinal Pigmented Epithelium as a Potential Therapy for Age-related Macular Degeneration

Authors: Bowen Wang[1]†, Li Wang[1]†, Sijie Gu[1], Yankun Yu[1], Huaxing Huang[1], Kunlun Mo[1], He Xu[2], Fanzhu Zeng[2], Yichen Xiao[1], Lulu Peng[1], Chunqiao Liu[1], Nan Cao[2], Yizhi Liu[1]*, Jin Yuan[1]*, Hong Ouyang[1]*

Affiliations:

[1]State Key Laboratory of Ophthalmology, Zhongshan Ophthalmic Center, Sun Yat-sen University, Guangzhou, China.

[2] Program of Stem Cells and Regenerative Medicine, Fifth Affiliated Hospital, Zhongshan School of Medicine, Sun Yat-Sen University, Guangdong, China.

*Corresponding author. Email: ouyhong3@mail.sysu.edu.cn (H.O); yuanjincornea@126.com. (J.Y); yizhi_liu@aliyun.com (Y.L)

†These authors contributed equally to this work.

This PDF file includes: Figures. S1 to S5

Other Supplementary Materials for this manuscript include the following:
Data S1: D609_RNASeq_TPM_Matrix.xls

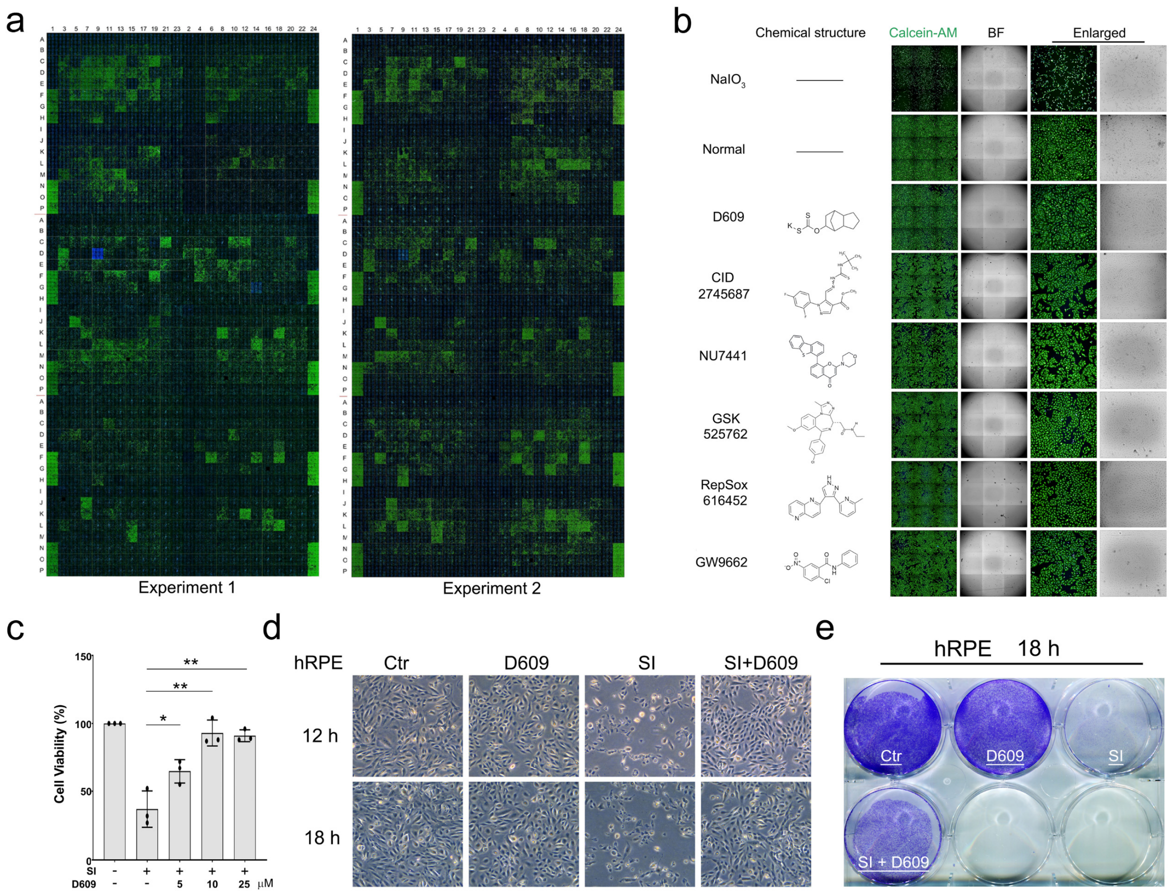

Figure S1. High-Content screening images of antioxidative damage compounds. (**a**) Calcein-AM/Hoechst images of the independent high-content screening experiments. (**b**) The chemical structures and Calcein-AM/Hoechst images of phase-contrast images of the ARPE-19 cells treated with compounds by preliminary screening, including D609, NU7441, GSK525762, CID 2745687, GW9662, and RepSox 616452. (**c**) ARPE-19 cells treated with D609 (5 μM, 10 μM or 25 μM), SI (10 μM), or combination for 18 hours, and cell viability tested by CCK8-kit. Data are shown as means ± SD. (**d**) Phase-contrast images of hRPE cells treated with D609 (10 μM) and/or SI (10 μM) at 12 or 18 hours. (**e**) Crystal violet staining after D609 (10 μM) and/or SI (10 μM) treatment in hRPE cells. *$p < 0.05$, **$p < 0.01$. n = 3.

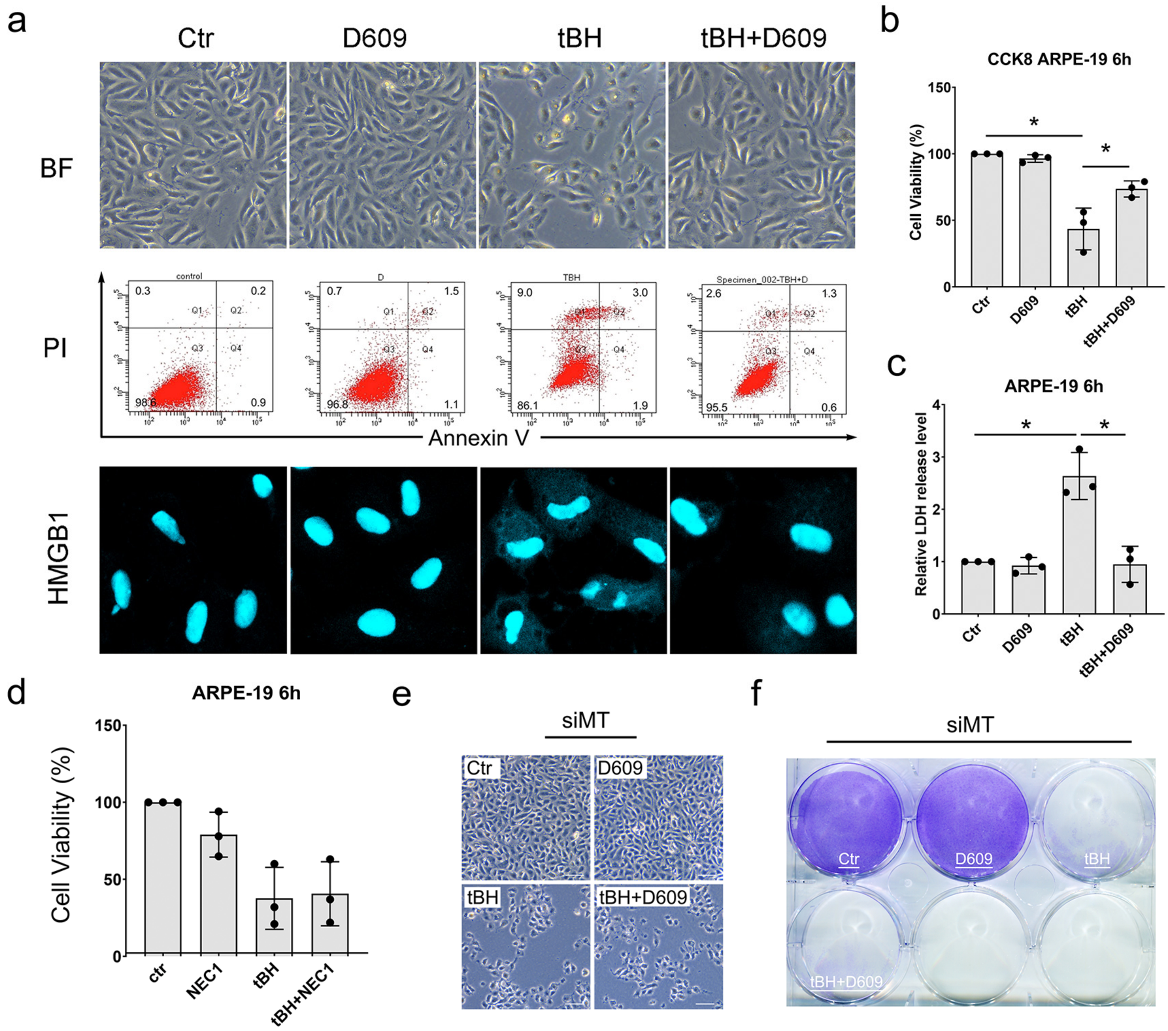

Figure S2. D609 inhibits tBH induced ARPE-19 cell death. (**a**) Upper panel: Phase-contrast images of ARPE-19 cells treated with D609 (10 μM), tBH (200 μM) or combination for 6 hours; Middle panel: Apoptotic cell death analyzed by flow cytometry using Annexin V and PI double-staining. ARPE-19 cells treated with D609, tBH, or a combination for 6 hours. Quadrant 1 and 2 (PI-positive) represent dead cells, quadrant 2 and 4 represent apoptotic cells; Lower panel: location and expression of HMGB1 in ARPE-19 cells treated with D609, tBH, or combination by immunofluorescence imaging. BF: bright field. (**b**) Cell viability of ARPE-19 cells treated with D609, tBH, or combination by CCK8-kit. Data are shown as means ± SD. (**c**) LDH release of ARPE-19 cells treated with D609and/or tBH. Data are shown as means ± SD. (**d**) Cell viability

(CCK8) of ftRPE cells treated with 30 μM of necroptosis inhibitor necrostatin-1 (Nec-1) and/or tBH (200 μM) or combination for 6 hours. Data are shown as means ± SD. (**e**) Phase-contrast bright field (BF) after tBH and/or D609 treatment (6 hours) in MT knockdown ARPE-19 cells. (**f**) crystal violet staining after tBH and/or D609 treatment (6 hours) in MT knockdown ARPE-19 cells. Data presented are averaged from 3 separate experiments. *$p < 0.05$. $n = 3$.

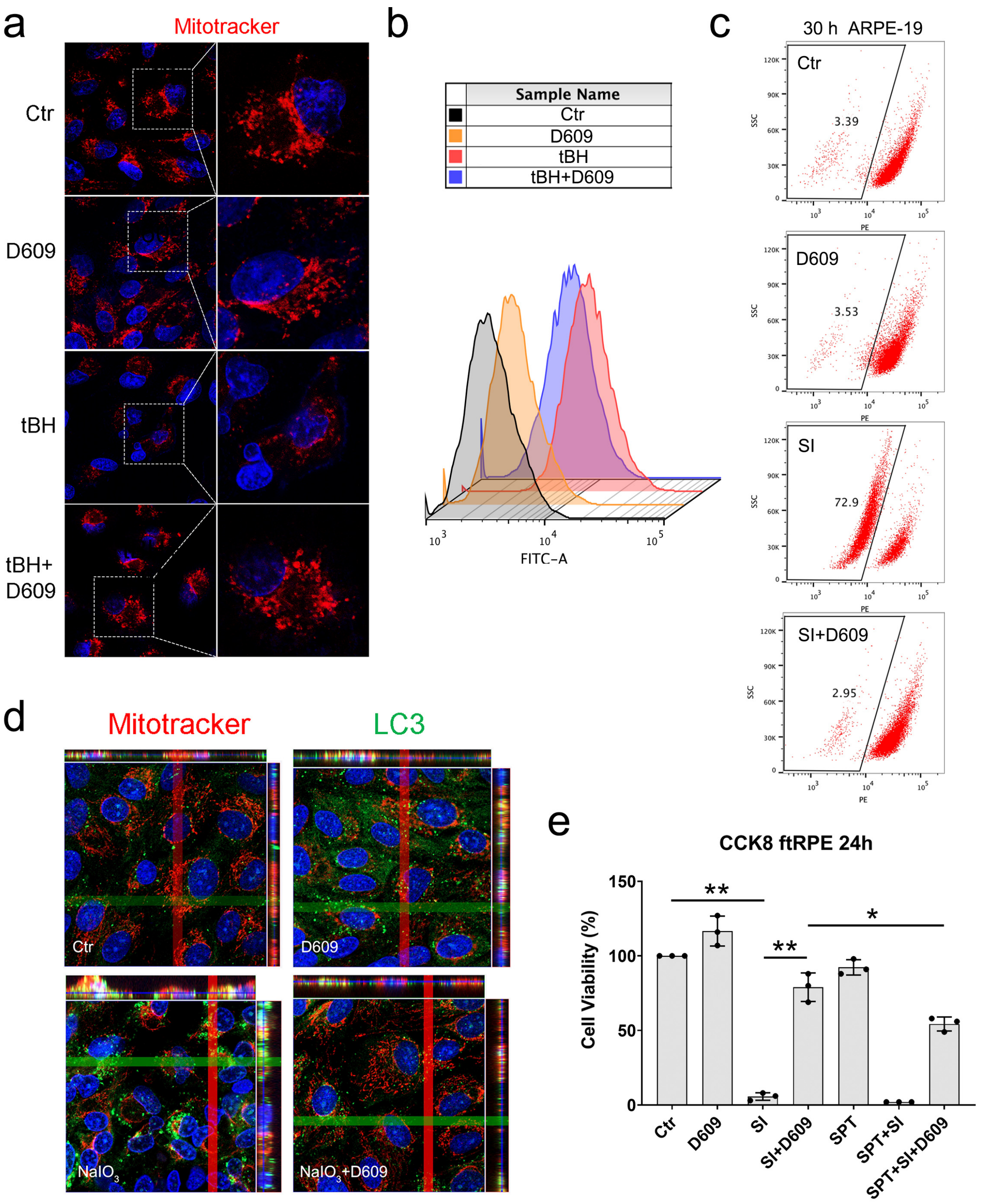

Figure S3. D609 inhibited mitochondrial dysfunction and ROS production caused by oxidative stress in ARPE-19 cells. (**a**) Mitotracker imaging represents the mitochondria mass loss after 6 hours tBH (200 μM) treatment in ARPE-19 cells, and the rescue effect of D609. (**b**) ROS

production level of ARPE-19 cells treated with 6 hours of tBH (200 μM), D609 (10 μM) or combination measured by flow cytometry. (**c**) MTMP measured in ARPE-19 cells treated with D609 (10 μM), SI (10 μM) or combination for 30 hours by flow cytometry. (**d**) 3-D reconstructed image demonstrated the colocalization of autophagosomes (LC3, green) of mitochondria (Mitotracker, red) generated from z-stack scanning. (**e**) Cell viability of ftRPE cells in the presence or absence of autophagy inhibitor Spautin-1 (10 μM) when treated with D609 (10 μM) and/or SI (10 μM) for 18 hours by CCK8 assay. Data are shown as means ± SD. Data presented are averaged from 3 separate experiments. *$p < 0.05$, **$p < 0.01$.

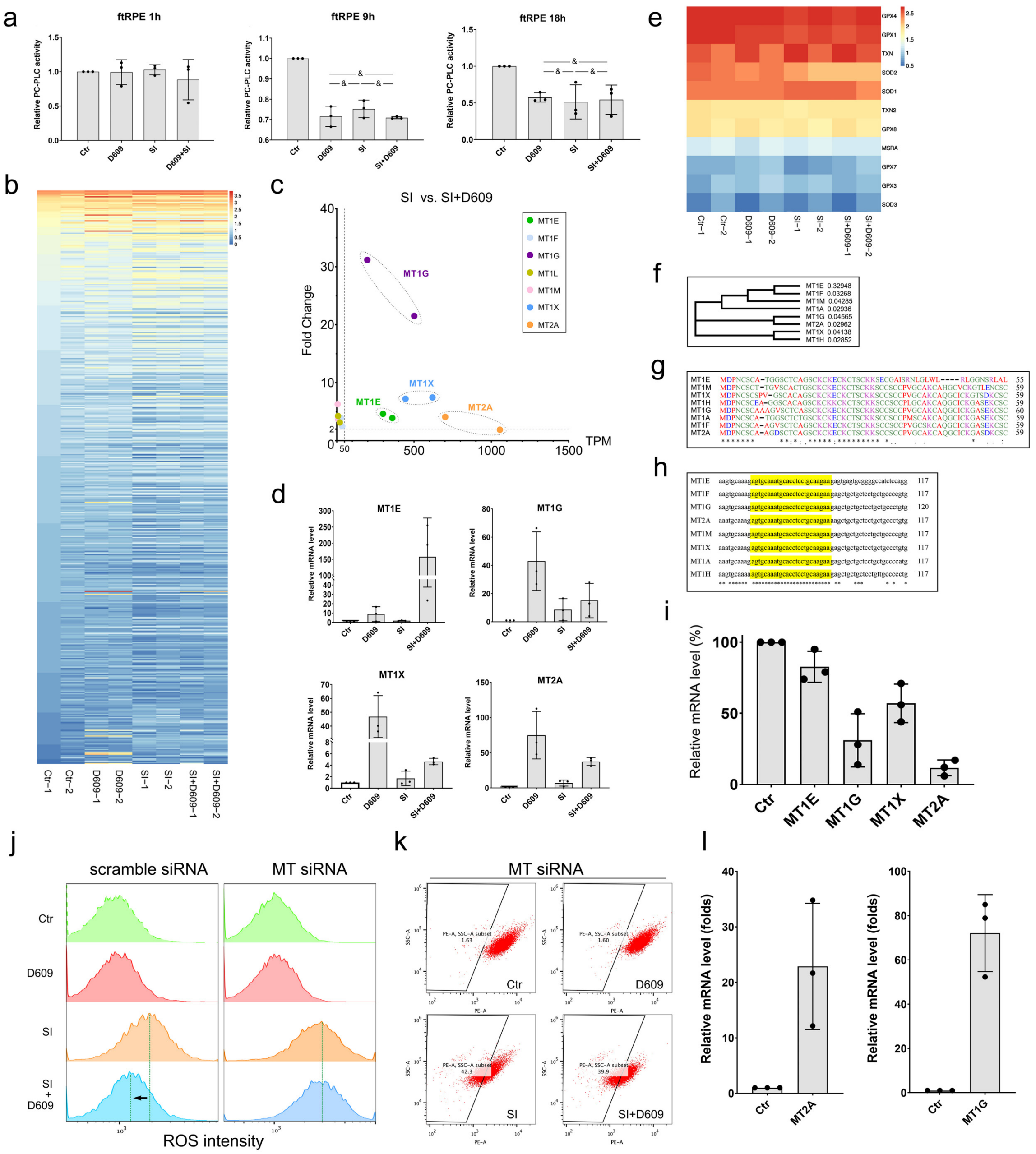

Figure S4. PC-PLC activity assay, RNA-Seq analysis and conserved region analysis of the MT protein family. (**a**) PC-PLC activity measured in ftRPE cells treated with D609 (10 µM), SI (10 µM) at 1, 9, or 18 hours. & p >0.1. Data are shown as means ± SD. (**b**) Heatmaps of the whole genome on the transcription level. (**c**) The expression fold change and expression level of metallothioneins in the SI-D609 cotreated group compared to the SI-treated group from ftRPE

RNA-seq data. TPM (transcripts per million) (**d**) qPCR result of metallothioneins in ftRPE cells treated with D609 (10 μM) and/or SI (10 μM) at 12 hours. Data are shown as means ± SD. (**e**) Heatmaps of antioxidant family genes on the transcription level. (log2 of normalized tpm). (**f**) Cluster dendrogram of the metallothionein gene family. (**g**) Amino acid sequence alignment of the metallothionein gene family. (**h**) Alignment of the metallothionein gene family, the highlighted area shows the conserved region of all metallothionein gene family. (**i**) qPCR result of metallothioneins in ftRPE cells treated with siRNA of metallothionein (siMT) for 48 hours, the Ctr is ftRPE cells treated with scrambled siRNA. Data are shown as means ± SD. (**j**) ROS production level of ARPE-19 cells treated with SI (10 μM) and co-treatment with D609 (10 μM) for 6 hours measured by flow cytometry upon MT or scramble siRNA interference. (**k**) Upon MT or scramble siRNA interference, Mitochondrial transmembrane potential (MTMP) measured in ARPE-19 cells treated with D609 (10 μM) and/or SI (10 μM) for 12 hours by flow cytometry of TMRE staining. (**l**) qPCR result of MT1G and MT2A upon overexpression in ARPE-19 cells by plasmids of MT2A (pcDNA3.1-MT2A-GFP), MT1G (pcDNA3.1-MT1G-GFP) and Ctr vector (pcDNA3.1-GFP) for 48 hours. Data are shown as means ± SD. Data presented are averaged from 3 separate experiments. * indicates conserved amino acid or nucleotide.

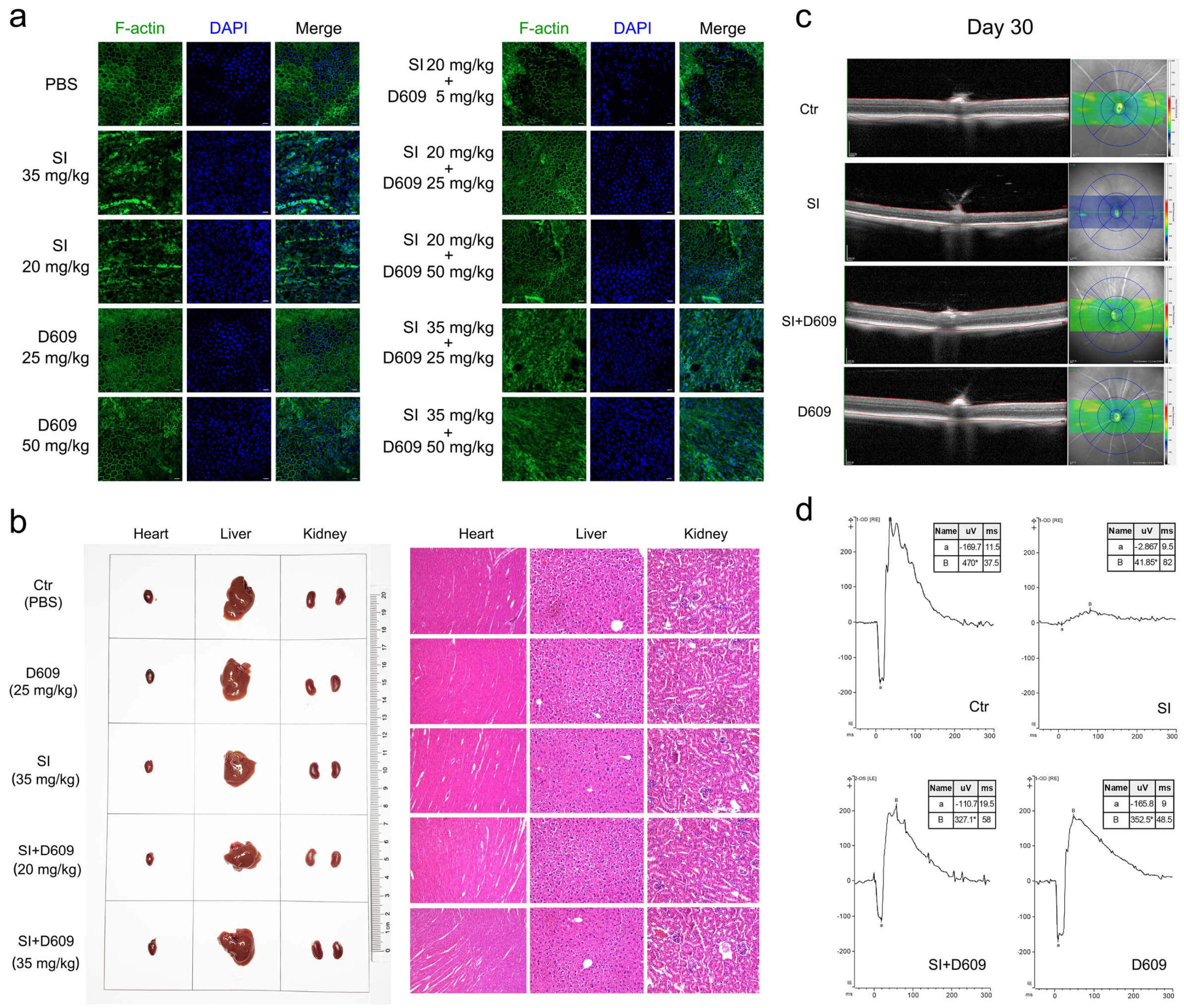

Figure S5. D609 injection dose determination, major organ toxicity, and physiological function alteration of post-injection in mice. (**a**) F-actin structure of day 7 post-injected mouse retina by phalloidin staining. (**b**) Images of the heart, liver, and kidney of mice at day 7 post-injected with D609 (25 mg/kg) and/or SI (20, 35 mg/kg). (**c**) Representative mouse OCT images of the central retina and retinal thickness at day 30 post-injected with D609 (25 mg/kg) and/or SI (20 mg/kg). (**d**) Mouse mesopic ERG responses (3cd.s.m^{-2}) recorded at day 30 post-injected with D609 (25 mg/kg) and/or SI (20 mg/kg); n = 5.
